# A Comparison of Presentations with Self-Harm to Hospital in Lithuania and Ireland

**DOI:** 10.3390/ijerph18052418

**Published:** 2021-03-02

**Authors:** Samah Kamal, Mark McGovern, Aida Kunideliene, Patricia Casey, Anne M. Doherty

**Affiliations:** 1 School of Medicine, Lithuanian University of Health Sciences, A Mickeviciaus g, 44307 Kaunas, Lithuania; samahkamal.abbas@hotmail.com; 2 Department of Psychiatry, University Hospital Galway, Newcastle, H91 YR71 Galway, Ireland; mark.mcgovern@hse.ie; 3 Department of Psychiatry, Kaunas Kilincos, Eiveniu g. 2, 50161 Kaunas, Lithuania; aida.Kunideliene@kaunoklinikos.ie; 4 Department of Liaison Psychiatry, Mater Misericordiae University Hospital, 63 Eccles Street, D07 YH5R Dublin 7, Ireland; profcasey@esatclear.ie; 5 Department of Psychiatry, University College Dublin, 63 Eccles Street, D07 YH5R Dublin 7, Ireland

**Keywords:** self-harm, suicide, depression, suicidal ideation, suicidal behaviours, psychiatry, mental health, Ireland, Lithuania

## Abstract

Suicide is a serious problem globally, especially in Europe, with suicide rates varying between different countries. Self-harm is a known risk factor for dying by suicide and represents an opportunity to intervene in order to treat any associated mental illness and reduce risk. This study aimed to compare the characteristics of people presenting to hospital with self-harm at two clinical sites: Galway, Ireland and Kaunas, Lithuania. Data were obtained from the services’ database and anonymised for analysis. Over a 5-month period, 89 patients presented with self-harm at the Lithuanian site and 224 patients presented with self-harm at the Irish site. This study found significant differences in presentation, diagnosis and treatment between the two sites. All patients at the Lithuanian site were admitted to psychiatry, compared to 22% of patients at the Irish site (*p* < 0.001). In Lithuania, the main clinical diagnoses were adjustment disorder (37.1%) and major depression (20.2%), compared to substance misuse being the main clinical diagnosis (33.8%) in Ireland (*p* < 0.001). There were significant differences in the prescription of psychotropic medications (which were three times more commonly prescribed at the Lithuanian site) after controlling for age, gender and psychiatric history (*p* < 0.001). Further research is required to understand the cultural context behind and further association between hospitalisation and future death by suicide.

## 1. Introduction

Suicide is the eighteenth leading cause of death internationally, with approximately 800,000 people dying each year [1]. Europe in particular has high rates of death by suicide, with 15.4 per 100,000 people dying by suicide per year on average. Lithuania has the world’s highest suicide rate: a rate of 31.9 per 100,000 [1].

Suicidal behaviours, including self-harm, may be regarded as complex socio-cultural phenomena, and are not always associated with mental illness [2,3,4]. Self-harm is defined as “any act of self-poisoning or self-injury carried out by an individual irrespective of motivation. This commonly involves self-poisoning with medication or self-injury by cutting. There are several important exclusions that this term is not intended to cover. These include harm to the self, arising from excessive consumption of alcohol or recreational drugs, or from starvation arising from anorexia nervosa, or accidental harm to oneself” [5,6].

The topic of suicide and suicide attempts is highly complex and involves a range of risk factors (including the male gender, socio-economic marginalisation, adverse childhood experiences, stigma, cultural factors and mental illness), which can pre-dispose an individual to mental illness and distress and are also associated with suicidal ideations, behaviours and even suicide. Men are known to have a significantly higher rate of suicide than women (who have higher rates of self-harm), and the rate of attempted suicides decreases with age (specifically in those who are over 65) while the rate of death by suicide increases [7]. A study of self-harm in adolescents found that 16.4% of the participants wished to die but had no other risk factors for suicide [8]. In addition, there are cultural variations in the expression of distress both within and without the context of mental illness [9,10].

Suicidal ideation and behaviours may be associated with various mental disorders, including major depression, adjustment disorders and borderline personality disorder [11,12,13,14]. Borderline personality disorder in particular carries a high risk of self-harm and suicidal behaviours, as well as an elevated risk of dying by suicide [14]. A study of patients with chronic depression and suicide attempts reported that 20% of participants had a previous suicide attempt [15]. A US-based study demonstrated the difficulties in attributing suicidal ideations and behaviours to any one mental illness, and reported that 80% of those with a history of suicidal behaviours had a previous mental illness [16]. Self-harm is a major risk factor for future death by suicide: a meta-analysis conducted by Carroll et al reported that 1 in 25 patients with a history of self-harm die by suicide within 5 years [17]. 

Substance abuse and alcohol abuse are associated with increased suicide ideation, suicide attempts and suicide [18]. Roche et al reported that alcohol consumption per capita is closely related to suicide rate and that the type of alcohol is also associated with suicide (i.e., they noted that wine consumption seemed to have no association with suicide, but higher spirit consumption per capita was associated with both male and female suicide rates and beer consumption per capita was only associated with male suicide rates) [19]. Some studies explored the relationship between previous suicide attempts and the method used by those who died by suicide. They found that those who have died by suicide with a history of prior suicide attempts were twice as likely to die by poisoning rather than firearms [20]. Research from Lithuania has shown an association between alcohol and self-harm: over 70% of male patients presenting with self-harm were identified as having problem-drinking [21].

An individual’s recent discharge from a psychiatric hospital is a risk factor for suicide, which is related to mental illness: multiple studies, including Irish studies, have indicated that the risk of suicide is higher after discharge from a psychiatric hospital [22]. A study published in 2017 showed that the post-discharge suicide rate was 484 per 100,000 individuals and that the risk of suicide is exceptionally higher in the 3-month period post-discharge. This study reported that the risk of dying by suicide post-discharge is higher in patients who were admitted due to suicidal ideas or self-harm [23]. 

Irish research in the area of self-harm found that variations in services available may lead to differences in assessment rates and the treatments offered; in particular, the availability of specialist mental health staff in the emergency departments and in the acute hospital may determine the proportion of patients who receive guideline-based care, and local bed availability will determine the threshold for psychiatric admission [24]. The variation in this practice in Ireland alone ranges from 10–74%, depending on the hospital [25].

Lithuania has a high suicide rate. Although few studies have been conducted on the relationship between suicide and self-harm in Lithuania, one study found a correlation between hanging as the method of suicide and socio-economic status [26]. In Ireland, a study examined hospital-treated self-harm and suicide in the west of Ireland. They discovered that the relationship between the suicide rate and para-suicidal behaviour in Ireland significantly varied by age, gender and marital status. They reported that the most commonly-used method of self-harm was drug overdose, and, like Lithuania, hanging was the most commonly used method of suicide [27].

Although both countries provide free mental health care, there are differences in the configuration of mental health services between Ireland and Lithuania. In Ireland, the bulk of mental healthcare is delivered on an outpatient basis, with relatively low admission rates. Ireland has one on the lowest rates of available psychiatric beds in Europe (23/100,000 (one-third of the European average of 70/100,000)), and mental health services only receive 6% of the health budget [28]. Since 2013, there has been an National Clinical Programme implemented to improve the management of people who present with self-harm to emergency departments (ED), which has developed key performance indicators, such as a minimal number of people leaving before assessment, the involvement of caregivers in assessment and the formulation of emergency care plans [29]. Lithuania, on the other hand, has nearly double the proportion of psychiatric beds (42/10,000). In the past 20 years, there has been a significant move towards the development of community-based mental health services. This has been further strengthened by the “Programme of Implementation of the Mental Health Strategy” in 2008 [30,31].

The aim of this study was to compare demographic variables, clinical characteristics and the psychiatric treatment of patients presenting with self-harm over a 5-month period from January–May 2019 at two centres: one in Ireland and the other in Lithuania. 

## 2. Materials and Methods

This is a cross-sectional study of patients presenting with self-harm (including those with suicidal intent) over a 5-month period from January–May 2019 at two hospitals: one in Lithuania and one in Ireland. We use the term “self-harm” throughout to include the full spectrum of self-injurious behaviours from non-suicidal self-injury to serious suicide attempts, as without any formal measure of intent it is inaccurate to describe these presentations as suicide attempts, although some of these presentations were indeed suicide attempts [5].

This study examined the characteristics of patients presenting to two university hospitals with self-harm. The Lithuanian site is a large (2000 bed) university hospital in the city of Kaunas (Kaunas Klinicos (KK)) and serves the local population; the city has a population of 328,763 people. The site in Ireland is a university hospital (850 beds) in the city of Galway (University Hospital Galway (UHG)), which has a population of 79,934 people. Both hospitals have the regional EDs on-site. The registries of all patients who presented to the ED with self-harm were examined at both sites.

The following demographic variables were collected at both sites: age, gender, primary clinical psychiatric diagnosis based on ICD-10, psychiatric history, method of self-harm and management strategy, including the psychotropic medications prescribed. 

The population selected in this study included patients who presented with self-harm from 1 January 2019 to 31 May 2019 in KK and UHG. This study utilised the database held by the liaison psychiatry team at UHG, which included all self-harm presentations to the service via the ED or from medical/surgical wards. This database is maintained to monitor service activity and needs. The data were anonymised, and episode of self-harm were separated out and analysed. Similar data were extracted from patient files at KK. 

Data were analysed with SPSS using Chi-square (χ^2^) tests to compare nominal variables between the two groups and *t*-tests or Mann–Whitney u-tests to compare scale variables. Logistic regression was conducted to examine differences between UHS and KK while controlling for confounding variables. The data were analysed as a whole, then analysed by site. Differences were considered statistically significant when *p* < 0.05.

Ethical approval from the Department of Bioethics and the Bioethics Committee at the Lithuanian University of Health Sciences (LUHS) was obtained. Ethical approval for the UHG arm of the study was granted by the Saolta Clinical Research Ethics Committee.

## 3. Results

In the 5-month period of this study, 89 patients presented with self-harm at KH, and 214 patients presented with self-harm at UHG. Further detail is provided in Table 1. 

At KK, the mean age was 40.6 years, and the majority of patients were male (n = 46; 51.7%). The majority of patients were referred from the ED, with only 5.6% and 10.1% referred from medical/surgical wards and critical care, respectively. The most common method of self-injury was poisoning, with 60 people utilizing that method (67.4%). Over three-quarters of these patients (78.7%) had a past psychiatric history. All patients who presented with self-harm (100%) had a previous psychiatric admission. At both sites, the diagnosis was made based on the initial full psychiatric assessment along with further montioring during inpatient admission if indicated. The majority of patients (95.5%) were prescribed a psychotropic medication (Table 2).

At UHG, the mean age of the patients was 31.2 years, and a majority of patients were female (59.8%). The majority (90.2%) were referred from the ED, with only 3.3% and 6.5% referred from medical/surgical wards and critical care, respectively. The most common method of self-injury was poisoning, with 122 people utilizing that method (57%). Over half of the patients (59.2%) had a past psychiatric history. One-fifth of patients who presented with self-harm (22%) had a psychiatric admission. Half of those patients presenting with self-harm (50.5%) were prescribed a psychotropic medication.

The patients presenting with self-injury at KK were significantly older, with a mean age over 9 years greater than the ages of those in UHG (*p* < 0.001). Although a majority of the patients in Kaunas were male (51.7%) and a majority of the patients in Galway were female (59.8%), this difference was not statistically significant (*p* = 0.066). There were no significant differences in the source of referral or the method of self-harm between the two sites. In Kaunas, patients presenting with self-harm were significantly more likely to have a psychiatric history than those presenting with self-harm at UHG (*p* = 0.001). There were significant differences in diagnosis between the two sites (*p* < 0.001): The most common diagnosis at KK was adjustment disorder, where it was diagnosed in 33 (37.1%) of patients presenting with self-harm. This was the fifth most common diagnosis at UHG (9%). The most common diagnosis at UHG was substance misuse disorder (33.8%), which was the joint fourth most common diagnosis at KK (7.9%). 

It is noteworthy that 86% of patients at the Galway site were assessed and discharged within 2 hours of referral to liaison psychiatry (or the psychiatric doctor on call during outside normal working hours). At KK, 95.5% patients were prescribed psychotropic medication, nearly double the proportion of patients prescribed psychotropic medication at UHG (50.5%) (*p* < 0.001). Patients at KK received a significantly greater number of medications (mean 1.73 (SD 0.8)) compared with mean 0.57 (SD 0.63) at UHG (*p* < 0.001). When those patients who were admitted to hospital only were examined at both sites, there were no significant differences between the two groups in terms of age, gender, referral source, psychiatric history or method of self-harm (Table 3). 

There were significant differences in primary psychiatric diagnosis and treatment (Table 4). A diagnosis of an active depressive episode was twice as common in the group admitted to UHG (39.3 %) as it was for the group admitted to KK (20.2). Adjustment disorder was the most common diagnosis in KK (37.1%), but no patients admitted with self-harm at UHG received this diagnosis (*p* < 0.001). In Kaunas, 95.5% of patients were prescribed psychotropic medication, nearly double the proportion of patients who received psychotropic medication at UHG (55.3%) (*p* < 0.001). Patients at KK received a significantly greater number of medications (1.73 per patient on average (SD 0.8)), compared to 0.66 per patient on average (SD 0.7) at UHG (*p* < 0.001). Patients at KK were significantly more likely to receive a prescription for an antipsychotic (*p* < 0.001) or a benzodiazepine (*p* < 0.001), that those at UHG.

When the differences between the two sites were examined using logistic regression after controlling for age, gender and psychiatric history, there were significant differences in the prescription of medications between KK compared with UHG as described in Table 5. (OR 3.02; *p* < 0.001; CI 0.334–1.414). 

## 4. Discussion

The results show significant differences between the patients presenting with self-harm at the two sites studied. KK patients were significantly older than the patients at UHG (*p* < 0.001). There was a significant difference in psychiatric history between the two sites: 78.7% of patients in KK had a previous psychiatric history, as compared to 59.2% of patients at UHG (*p* < 0.001). These differences were no longer significant when the UHG patients who were not admitted to psychiatry were excluded. The differences in patient management were clear—all patients at KK were admitted to psychiatry, compared with 22% of patients at UHG. Patients at KK were significantly more likely to be prescribed medication, a difference that remained significant after logistic regression. 

The literature has established that a previous psychiatric history is an important risk factor for suicide, with diagnoses of major depression, adjustment disorder, with borderline personality disorder and substance misuse being particularly prominent risk factors [2,3,4]. The findings of this study were congruent with those of the literature, although the prevalence of each condition differed significantly between the two sites: adjustment disorder, the most common diagnosis at KK (37.1%), was only diagnosed in 9% in the UHG cohort, and fell to 0% in the UHG population admitted to psychiatry. Substance misuse was the most common primary diagnosis in UHG (33.8%), but only represented 7.9% of the KK population’s diagnoses. This is surprising given the established association between alcohol and self-harm in previous Lithuanian research [21]. Our database captured the primary diagnosis and did not include any details on secondary diagnoses: it is possible that substance misuse may have been a secondary diagnosis in some of these cases. This difference in diagnosis can be due to many factors, including the history of the patients, different systems of health care and even the difference in culture between the two countries. 

It is worth noting that the prevalence of depressive episodes was similar at the two sites and was the second leading diagnosis at each: 20.2% of the diagnoses involved depressive episodes at KK and 20.3% of the diagnoses involved depressive episodes at UHG (rising to 39.3% of the UHG cohort who were admitted to psychiatry, where it was the most common diagnosis). Substance misuse is an established risk factor for self-harm and suicidal behaviours, so finding high amounts of this diagnosis are congruent with the literature [13,32,33,34]. 

This study found no significant difference in the method of self-harm used by patients between the two hospitals. In both sites, the majority of patients used poisoning as a method of suicide. A smaller number of patients used more potentially lethal methods such as hanging or drowning (approximately 11% at each site). Previous studies of methods used in self-harm compared the potential lethality of each method and the degree of suicidal intent. They reported that methods which are associated with greater potentially lethality were associated with higher levels of suicidal intent [35]. Certain high-lethality methods of self-harm such as hanging and jumping from a large height have an association with future death by self-harm [36,37,38].

At both sites, the main source of referral was through the ED. However, the rate of referrals from the critical care unit at KK was higher than the rate of referrals from the critical care units at UHG, which may indicate a greater severity of suicidal intent for patients at KK. Patients who require treatment in critical care units are likely to have died without treatment and may have more similarities to people who die due to self-harm rather than to those who may be discharged from the emergency department with no physical treatment required [39].

At KK, there was a 100% admission rate, while at UHG the rate of admission was 22%. These may represent radically different policies and reflect the differences in the delivery of mental health care in the two countries. It may also suggest that the patients presenting to KK are more severely unwell. A recent study showed that the risk of suicide is higher in the 3-month period post-discharge [23]. It is difficult to understand whether this has any relationship to the suicide rates in the two countries: it may be that a blanket policy to admit everyone for a period of time following an episode of self-harm at KK is in response to the country’s high suicide rates. Ireland’s number of mental health beds have dropped to 25% of the number of beds available in 2015, and, as a result, admission is only an option for people with severe mental illnesses [40]. When patients who were not admitted at UHG were omitted from the analysis, many of the differences between the two groups were no longer significant, suggesting the patients who were admitted at both sites may have represented more similar levels of illness severity. In addition to the very different admission policies, there are differences in the configuration of services across the two countries. Ireland has changed from historically having very large numbers of patients in psychiatric facilities to having one of the lowest rates of psychiatric bed availability in Europe [28]. Lithuania has undergone changes in the delivery of psychiatric services in recent decades, with significant reductions in the durations of admissions and of inpatient beds alongside the creation of community-based treatment programmes [28,30,31,41]. There are also differences in the prevalence of suicide and substance misuse across the two countries [1,21].

The prescription of medications (either on this assessment or pre-existing prescriptions) varied significantly between the two sites. The percentage of patients who were prescribed psychotropic medications at KK was almost double that at UHG. At KK, the most prescribed medications were antipsychotics (68.5%), followed by benzodiazepines (52.8%) and antidepressants (49.4%). Meanwhile at UHG, antidepressants were the most commonly prescribed medications (34.1%, which is still lower than that at KK), and only 3.3% of patients were prescribed benzodiazepines. This result shows the significant difference in the prescription of medications between the two sites and may reflect the differences in diagnosis between the two sites and the difference in the degree of associated psychopathology. It also may highlight the differences in the management of mental disorders between the two countries. The ready availability of medications may be seen as providing an easy method of self-harm; however, there is some evidence that being prescribed psychotropics may reduce self-harm, via a reduction in alcohol ingestion [42]. 

The limitations of this study include the difference in language at each site (Lithuanian at KK and English at UHG). Another limitation was the lack of information regarding previous suicide attempts and substance abuse. Seeing as this was a retrospective study, it was not possible to obtain a measure of suicidal intent, and, therefore, we have included all presentations with self-harm, regardless of intent. This is a database study, which limits the available data: for example, it does not include data regarding which members of the multi-disciplinary team provided input (e.g., psychological therapies) and there are no data on secondary diagnoses, nor does it include details of patients who died of their injuries after arrival in hospital (the databases included only those referred for a psychiatric opinion). This study does not capture the delivery of psychological therapies, as these would usually be referred through community or outpatient services. This is the first study to examine the differences in the patterns of self-harm between Lithuania and Ireland. 

Further research is needed to further explore the variables associated with self-harm: we need prospective studies which include measures of intent and which follow patients presenting with self-harm over years (and decades) to determine their clinical outcomes. There is a need for research to incorporate patient-reported outcomes, and to capture in detail the cultural and social factors that may help improve understanding of the differences in the presentation of mental illness and distress across different cultures and countries. 

## 5. Conclusions

The two sites in two different European Union countries showed significant differences in the diagnosis and treatment of self-harm. It is difficult to comment on the degree to which the culture or the clinical approach to the management of mental health difficulties may contribute to the differential in suicide rate between the two countries. 

## Figures and Tables

**Table 1:** Basic demographic & clinical details of patients presenting with self-harm at the two sites.

		Kaunas	Galway	*p*-Value
**Age in Years, Mean (SD)**		**40.6 (17.8)**	**31.2 (14.8)**	**<0.001 *^1^***
Gender	Female, n (%)	43 (48.3)	128 (59.8)	0.066 ^2^
	Male, n (%)	46 (51.7)	86 (40.2)	
Source of referral	Emergency Department (ED), n (%)	75 (84.3)	193 (90.2)	0.336 ^2^
	Medical/surgical wards, n (%)	5 (5.6)	7 (3.3)	
	Critical care, n (%)	9 (10.1)	14 (6.5)	
Method of self-harm	Poisoning, n (%)	60 (67.4)	122 (57)	0.083 ^2^
	Cutting, n (%)	18 (20.2)	59 (27.6)	
	Drowning, n (%)	1 (1.1)	11 (5.1)	
	Hanging, n (%)	9 (10.1)	13 (6.1)	
	Other, n (%)	1 (1.1)	9 (4.2)	
**Psychiatric history, n (%)**		**70 (78.7)**	**106 (59.2)**	**0.001 ^2^**
**Diagnosis**	**Depression, n (%)**	**18 (20.2)**	**27 (20.3)**	**<0.001 ^2^**
	**Adjustment disorder, n (%)**	**33 (37.1)**	**12 (9)**	
	**Personality disorder, n (%)**	**3 (3.4)**	**24 (18)**	
	**Substance misuse, n (%)**	**7 (7.9)**	**45 (33.8)**	
	**Anxiety disorder, n (%)**	**7 (7.9)**	**16 (12)**	
	**SMI (Psychosis or BPAD), n (%)**	**15 (16.9)**	**5 (3.8)**	
	**Other, n (%)**	**6 (6.7)**	**4 (3.0)**	
**Admission rate to psychiatric unit, n (%)**		**89 (100)**	**47 (22)**	**<0.001 ^2^**

^1^ Independent Sample T-test; ^2^ Chi Square test. Significant values in bold typeface. SMI = severe mental illness; BPAD = bipolar affective disorder.

**Table 2:** A comparison between the prescription rate of psychotropic medications at the two sites.

	Kaunas	Galway	*p*-Value
**Medications, n (%)**	**85 (95.5)**	**108 (50.5)**	**<0.001** ^2^
*Medication type*			
**Antidepressant, n (%)**	**44 (49.4)**	**73 (34.1)**	**0.009** ^2^
Mood stabiliser, n (%)	3 (3.4)	13 (6.1)	0.257 ^2^
**Antipsychotic, n (%)**	**61 (68.5)**	**27 (12.6)**	**<0.001** ^2^
**Benzodiazepine, n (%)**	**47 (52.8)**	**7 (3.3)**	**<0.001** ^2^
Antihistamine, n (%)	0	2 (0.9)	0.498 ^2^
**Number of medications, mean (SD)**	**1.73 (0.822)**	**0.57 (0.63)**	**<0.001 *^1^***

^1^ Independent Sample T-test; ^2^ Chi Square. Significant values in bold typeface.

**Table 3:** Basic demographic and clinical details of patients presenting with self-harm who were admitted to a psychiatric unit at the two sites.

		Kaunas n = 89	Galway n = 47	*p*-Value
**Age in Years, Mean (SD)**		40.6 (17.8)	38.6 (16.2)	0.528 *^1^*
Gender	Female, n (%)	43 (48.3)	22 (46.8)	0.876 ^2^
	Male, n (%)	46 (51.7)	25 (53.2)	
Source of referral	ED, n (%)	75 (84.3)	42 (89.4)	0.709 ^2^
	Medical/surgical wards, n (%)	5 (5.6)	2 (4.3)	
	Critical care, n (%)	9 (10.1)	3 (6.4)	
Method of self-harm	Poisoning, n (%)	60 (67.4)	33 (70.2)	0.07 ^2^
	Cutting, n (%)	18 (20.2)	4 (8.5)	
	Drowning, n (%)	1 (1.1)	2 (4.3)	
	Hanging, n (%)	9 (10.1)	4 (8.5)	
	Other, n (%)	1 (1.1)	4 (8.5)	
Psychiatric history, n (%)		70 (78.7)	27 (71.1)	0.356 ^2^
**Diagnosis**	**Depressive episode, n (%)**	**18 (20.2)**	**11 (39.3)**	**0.001** ^2^
	**Adjustment disorder, n (%)**	**33 (37.1)**	**0 (0)**	
	**Personality disorder, n (%)**	**3 (3.4)**	**6 (21.4)**	
	**Substance misuse, n (%)**	**7 (7.9)**	**5 (17.9)**	
	**Anxiety disorder, n (%)**	**7 (7.9)**	**1 (3.6)**	
	**SMI (Psychosis/ BPAD), n (%)**	**15 (16.9)**	**3 (10.7)**	
	**Other, n (%)**	**6 (6.7)**	**2 (7.1)**	

^1^ Independent Sample T-test; ^2^ Chi Square. Significant values in bold typeface. Scheme 22. in Galway (*p* < 0.001).

**Table 4:** The prescription of psychotropic medications at the two sites for patients admitted to a psychiatric unit.

	Kaunas	Galway	*p*-Value
**Medications, n (%)**	**85 (95.5)**	**25 (55.3)**	**<0.001 ^2^**
Medication type			
**Antidepressant, n (%)**	44 (49.4)	17 (36.2)	0.139 ^2^
Mood stabiliser, n (%)	3 (3.4)	4 (8.5)	0.197 ^2^
**Antipsychotic, n (%)**	**61 (68.5)**	**7 (14.9)**	**<0.001 ^2^**
**Benzodiazepine, n (%)**	**47 (52.8)**	**3 (6.4)**	**<0.001 ^2^**
Antihistamine, n (%)	0	0	
**Number of medications, mean (SD)**	**1.73 (0.822)**	**0.66 (0.7)**	**<0.001 *^1^***

^1^ Independent Sample T-test, ^2^ Chi Square tests. Significant values in bold typeface.

**Table 5:** Comparison of the co-variates of self-harm by logistic regression, with site as dependant variable.

	Odds Ratio	*p*-Value	CI
**Age**	**27.8**	**<0.001**	**0.946–0.983**
Gender	2.1	0.143	0.853–3.007
**Medications**	**3.02**	**<0.001**	**0.017–0.143**
Psychiatric history	2.6	0.308	0.334–1.414

Significant values in bold typeface.

## Data Availability

Data may be provided upon reasonable request to the corresponding author.

## References

[1] WHO (2019). Suicide in the World: Global Health Estimates.

[2] Snowdon J. (2018). Differences between patterns of suicide in East Asia and the West. The importance of sociocultural factors. Asian J. Psychiatry.

[3] Brent D.A. (1995). Risk factors for adolescent suicide and suicidal behavior: Mental and substance abuse disorders, family environmental factors, and life stress. Suicide Life Threat. Behav..

[4] Phillips M.R. (2010). Rethinking the role of mental illness in suicide. Am. J. Psychiatry.

[5] NICE (2011). Self-Harm in over 8s: Lng Term Management.

[6] NICE (2013). Self-Harm. Quality Standard [QS34].

[7] De Leo D., Padoani W., Scocco P., Lie D., Bille-Brahe U., Arensman E., Faria S. (2001). Attempted and completed suicide in older subjects: Results from the WHO/EURO Multicentre Study of Suicidal Behaviour. Int. J. Geriatr. Psychiatry.

[8] Duarte T.A., Paulino S., Almeida C., Gomes H.S., Santos N., Gouveia-Pereira M. (2020). Self-harm as a predisposition for suicide attempts: A study of adolescents’ deliberate self-harm, suicidal ideation, and suicide attempts. Psychiatry Res..

[9] O’Keefe V.M., Tucker R.P., Cole A.B., Hollingsworth D.W., Wingate L.R. (2018). Understanding Indigenous Suicide Through a Theoretical Lens: A Review of General, Culturally-Based, and Indigenous Frameworks. Transcult Psychiatry.

[10] Goodmann D.R., Daouk S., Sullivan M., Cabrera J., Liu N.H., Barakat S., Leykin Y. (2020). Factor analysis of depression symptoms across five broad cultural groups. J. Affect. Disord..

[11] Doherty A.M., Jabbar F., Kelly B.D., Casey P. (2014). Distinguishing between adjustment disorder and depressive episode in clinical practice: The role of personality disorder. J. Affect. Disord..

[12] Casey P., Jabbar F., O’Leary E., Doherty A.M. (2015). Suicidal behaviours in adjustment disorder and depressive episode. J. Affect. Disord..

[13] Adhikari K., Metcalfe A., Bulloch A.G.M., Williams J.V.A., Patten S.B. (2020). Mental disorders and subsequent suicide events in a representative community population. J. Affect. Disord..

[14] Söderholm J.J., Socada J.L., Rosenström T., Ekelund J., Isometsä E.T. (2020). Borderline Personality Disorder with Depression Confers Significant Risk of Suicidal Behavior in Mood Disorder Patients—A Comparative Study. Front. Psychiatry.

[15] Ernst M., Kallenbach-Kaminski L., Kaufhold J., Negele A., Bahrke U., Hautzinger M., Leuzinger-Bohleber M. (2020). Suicide attempts in chronically depressed individuals: What are the risk factors?. Psychiatry Res..

[16] Nock M.K., Hwang I., Sampson N.A., Kessler R.C. (2010). Mental disorders, comorbidity and suicidal behavior: Results from the National Comorbidity Survey Replication. Mol. Psychiatry.

[17] Carroll R., Metcalfe C., Gunnell D. (2014). Hospital presenting self-harm and risk of fatal and non-fatal repetition: Systematic review and meta-analysis. PLoS ONE.

[18] Norström T., Rossow I. (2016). Alcohol Consumption as a Risk Factor for Suicidal Behavior: A Systematic Review of Associations at the Individual and at the Population Level. Arch. Suicide Res..

[19] Roche S.P., Rogers M.L., Pridemore W.A. (2018). A cross-national study of the population-level association between alcohol consumption and suicide rates. Drug Alcohol. Depend..

[20] Jamison E.C., Bol K.A. (2016). Previous Suicide Attempt and Its Association With Method Used in a Suicide Death. Am. J. Prev. Med..

[21] Dambrauskiene K., Adomaitiene V., Zalinkevicius R., Jariene G., Vilkas V., Rybakova I., Dunderiene L. (2019). Can Suicide Attempt be Related to Problem Drinking: Cohort Study. Alcohol Alcohol..

[22] Perry I.J., Corcoran P., Fitzgerald A.P., Keeley H.S., Reulbach U., Arensman E. (2012). The incidence and repetition of hospital-treated deliberate self harm: Findings from the world’s first national registry. PLoS ONE.

[23] Chung D.T., Ryan C.J., Hadzi-Pavlovic D., Singh S.P., Stanton C., Large M.M. (2017). Suicide Rates after Discharge from Psychiatric Facilities: A Systematic Review and Meta-analysis. JAMA Psychiatry.

[24] Griffin E., Gunnell D., Corcoran P. (2020). Factors explaining variation in recommended care pathways following hospital-presenting self-harm: A multilevel national registry study. BJPsych Open..

[25] Carroll R., Corcoran P., Griffin E., Perry I., Arensman E., Gunnell D., Metcalfe C. (2016). Variation between hospitals in inpatient admission practices for self-harm patients and its impact on repeat presentation. Soc. Psychiatry Psychiatr. Epidemiol..

[26] Starkuviene S., Kalediene R., Petrauskiene J. (2006). Epidemic of suicide by hanging in Lithuania: Does socio-demographic status matter?. Public Health.

[27] Corcoran P., Keeley H.S., O’Sullivan M., Perry I.J. (2003). Parasuicide and suicide in the south-west of Ireland. Ir. J. Med. Sci..

[28] WHO (2017). Monitoring Mental Health Systems and Services in the WHO European Region.

[29] CPsychI H. (2016). National Clinical Programme for the Assessment and Management of Patients Presenting to Emergency Departments Following Self-Harm.

[30] European Commission (2017). Mental Health Briefing Sheets: Facts and Activities in Member States—Lithuania.

[31] Puras D. (2005). Mental health in Lithuania. Int. Psychiatry.

[32] Singhal A., Ross J., Seminog O., Hawton K., Goldacre M.J. (2014). Risk of self-harm and suicide in people with specific psychiatric and physical disorders: Comparisons between disorders using English national record linkage. J. R. Soc. Med..

[33] Chesney E., Goodwin G.M., Fazel S. (2014). Risks of all-cause and suicide mortality in mental disorders: A meta-review. World Psychiatry.

[34] Mars B., Heron J., Klonsky E.D., Moran P., O’Connor R.C., Tilling K., Wilkinson P., Gunnell D. (2019). Predictors of future suicide attempt among adolescents with suicidal thoughts or non-suicidal self-harm: A population-based birth cohort study. Lancet Psychiatry.

[35] Haw C., Casey D., Holmes J., Hawton K. (2015). Suicidal Intent and Method of Self-Harm: A Large-scale Study of Self-Harm Patients Presenting to a General Hospital. Suicide Life Threat. Behav..

[36] Hawton K., Bergen H., Kapur N., Cooper J., Steeg S., Ness J., Waters K. (2012). Repetition of self-harm and suicide following self-harm in children and adolescents: Findings from the Multicentre Study of Self-harm in England. J. Child Psychol. Psychiatry.

[37] Bergen H., Hawton K., Waters K., Ness J., Cooper J., Steeg S., Kapur N. (2012). How do methods of non-fatal self-harm relate to eventual suicide?. J. Affect. Disord..

[38] Carroll R., Thomas K.H., Bramley K., Williams S., Griffin L., Potokar J., Gunnell D. (2016). Self-cutting and risk of subsequent suicide. J. Affect. Disord..

[39] Mohan C., Tembo V., McNicholas B., Doherty A.M. (2020). Defining high risk by clinical lethality: The different characteristics and management of the survivors of serious self-injury admitted to critical care, compared with lower lethality self-injury. Gen. Hosp. Psychiatry.

[40] Oireachtas H. (2018). Joint Committee on the Future of Mental Health Care: Final Report.

[41] Puras D., Germanavicius A., Povilaitis R., Veniute M., Jasilionis D. (2004). Lithuania mental health country profile. Int. Rev. Psychiatry.

[42] Chitty K.M., Dobbins T., Dawson A.H., Isbister G.K., Buckley N.A. (2017). Relationship between prescribed psychotropic medications and co-ingested alcohol in intentional self-poisonings. Br. J. Psychiatry.

